# Toward predictable universal genetic circuit design

**DOI:** 10.1002/qub2.48

**Published:** 2024-04-30

**Authors:** Yuanli Gao, Baojun Wang

**Affiliations:** ^1^ College of Chemical and Biological Engineering Zhejiang University Hangzhou China; ^2^ ZJU‐Hangzhou Global Scientific and Technological Innovation Center Zhejiang University Hangzhou China; ^3^ School of Biological Sciences University of Edinburgh Edinburgh UK

**Keywords:** circuit predictability, genetic circuit design, host independent, part characterization, portability

In the past 2 decades, synthetic biologists have applied systematic engineering principles to genetic circuit design to devise biological systems with bespoke behaviors, such as Boolean logic gates, signal filters, oscillators, state machines, perceptrons, and genetic controllers [1, 2]. Following a bottom‐up strategy, the genetic circuits are designed by assembling a set of well‐characterized biological components, or genetic parts [3], and optimized through the iterative Design‐Build‐Test‐Learn (DBTL) cycles. This design process has been accelerated by computational tools to predict parts’ functionality in different contexts and design automation platforms to concatenate parts according to user‐defined specifications [4, 5].

However, the scope of these remarkable achievements has been confined to a few model organisms, such as *Escherichia coli* and *Saccharomyces cerevisiae*, which have served as the primary platforms for demonstrating novel regulatory modalities and enabling tools. While these organisms have indeed showcased their potential for real‐world applications (e.g., *E. coli* Nissle 1917‐based bacterial therapeutics against cancer and metabolic disorder [6]), other non‐model organisms, with unique metabolisms (e.g., precursor turnover, product secretion, and tolerance to extreme conditions) and adaptations for specific environments (e.g., human organs, tumors, soil, and ocean), harbor immense potential for a myriad of unexplored applications [7]. Therefore, transferring the genetic parts and tools to non‐model organisms presents a thrilling opportunity to transform genetic circuits from laboratory to field and extend their applications far beyond the traditional boundaries.

The path to harnessing the potential of non‐model organisms is challenging due to distinct host contexts. The regulatory modalities developed in model chassis cannot be readily ported into a new chassis with their initial functionality, as they must adapt to host‐specific gene expression machinery, metabolism, and even the DNA vectors used to encode the genetic circuits (Figure 1A). Consider the case of the human commensal bacterium *Bacteroides thetaiotaomicron*. The RNA polymerase (RNAP) σ^70^ factor recognizes a −33/−7 consensus sequence in *B*. *thetaiotaomicron* instead of the −35/−10 consensus sequence in *E. coli*, precluding the direct transplantation of constitutive promoter libraries between these organisms [10] (① in Figure 1A). The anaerobic conditions used to culture *B. thetaiotaomicron* also compromise the functionality of genetic parts requiring oxygen, such as fluorescent proteins (② in Figure 1A). Additionally, the genome of *B. thetaiotaomicron* is often used as the DNA vector for integrating exogenous genetic circuits, while those in *E. coli* are usually encoded in plasmids. This difference in copy numbers leads to the failure of directly transferring the typical transcription repressor‐based promoter architectures from *E. coli* to *B. thetaiotaomicron* [10, 11] (③ in Figure 1A), necessitating meticulous efforts to find optimal operator numbers in a promoter or proper DNA‐binding proteins to debug these circuits [10, 11]. These challenges underscore the need for a universal genetic circuit that can function robustly and predictably across diverse organisms.

**FIGURE 1:**
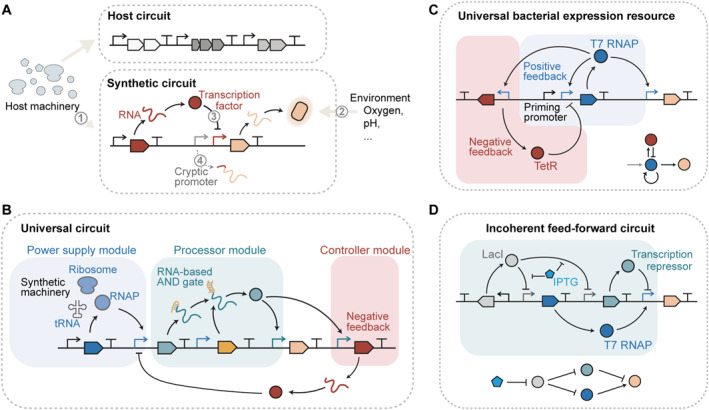
Universal genetic circuits that function robustly in diverse host backgrounds. (A) Host contexts render the performance of synthetic circuits unpredictable. Different gene expression machinery (①) and environmental conditions (②) preclude the direct transplantation of some part categories across diverse hosts. The copy number variations (③) of DNA vectors affect the function of transcription factors. The cryptic promoters (④) that emerged from part assembly are difficult to identify in non‐model organisms. (B) A universal circuit comprises three modules: a power supply module encoding synthetic gene expression machinery, a processor module determining input‐output relationships, and a controller module decoupling synthetic circuits from variations in host contexts. (C) The design of the universal bacterial expression resource [8]. A priming promoter drives the expression of T7 RNAP, which self‐activates through a positive feedback loop and triggers the expression of TetR for negative feedback. (D) The design of the incoherent feed‐forward circuit described by Qin et al. [9]. The addition of IPTG evokes the expression of T7 RNAP and transcription repressor, which activates and represses target gene expression, respectively. IPTG, isopropyl β‐D‐1‐thiogalactopyranoside; RNAP, RNA polymerase; TetR, tetracycline repressor.

Ideally, the universal genetic circuit should be insulated from host contexts, including the extracellular, cellular, and genetic contexts [1]; that is, it should be decoupled from specific biological processes, independent of particular resources (e.g., host cofactor and oxygen), orthogonal to host machinery, and resilient to copy number variations. Therefore, a universal genetic circuit needs to comprise several modules: a power supply module that encodes heterologous gene expression machinery (e.g., RNAP) orthogonal to host machinery; a processor module that programs the input‐output relationship for precise control of target gene expression; a controller module that decouples synthetic circuits from variations in host contexts (Figure 1B).

Such a universal circuit was first attempted by Kushwaha and Salis in 2015 [8]. In their device called the universal bacterial expression resource (UBER), a cross‐species priming promoter drives the expression of T7 RNAP, which self‐activates through a positive‐feedback loop and triggers the expression of tetracycline repressor (TetR) transcription repressors for self‐repression (Figure 1C). The positive feedback loop decouples the expression of T7 RNAP from the ambiguous transcription rates of the priming promoter in different bacterial species, and the TetR‐based negative feedback loop prevents the overexpression of T7 RNAP to eliminate its toxicity. The UBER circuit was functional in multiple bacterial species, namely *E. coli*, *Pseudomonas putida*, and *Bacillus subtilis* [8]. However, this system remains a proof‐of‐concept demonstration, as it lacks precise quantitative analysis and comparison of the circuits’ performance in different chassis, rendering its functionality poorly predictable when ported across the bacterial domain. Furthermore, this system lacks variants to T7 RNAP and TetR, rendering it hard to program the processor module to carry out complex tasks such as logic computation.

In this issue of *Quantitative Biology*, Qin et al. [9] have made significant strides in tackling these limitations. They systematically investigated the parameters of genetic circuits based on T7 RNAP and a set of transcription repressors (TetR family repressors and bacteriophage repressors) in four different host microbes: *Streptomyces albus*, *Corynebacterium glutamicum*, *Pseudomonas entomophila*, and *E. coli*. They revealed the highly linear relationships of the relative T7 promoter activities across the four hosts and determined the hill coefficients and dissociation constants as host‐independent parameters of T7 RNAP and repressors. This rigorous part parametrization has then allowed for the precise, predictable design of Boolean logic gates (BUFFER and NOT gates) and complex analog circuits (incoherent feed‐forward circuits) in *C. glutamicum* (Figure 1D). This work provides a methodological reference for evaluating the cross‐species usability of transcription regulatory modules and offers insights into the parameters behind the transferability of these universal genetic parts. These findings pave the way to predictably design and tune a universal genetic circuit with complex functions and hold vast potential for realizing design automation of universal circuits in diverse hosts. The authors also reported an interesting phenomenon: cryptic promoters that could be recognized by the host transcription machinery formed from the composition of T7 promoters with repressor operators (④ in Figure 1A). This raised another concern: the emergence of cryptic promoters from part assembly is difficult to predict in non‐model organisms as few computational workflows are available for sequence‐to‐function prediction of promoters and the design of biological neutral sequences without additional transcriptional activities.

Future progress toward designing universal genetic circuits with ease needs to be made in several directions. First, the toolkit of the universal parts should be expanded by discovering portable part categories and establishing part libraries for orthogonal regulation at multiple levels of the central dogma. T7 RNAP has been crucial in driving gene expression in universal circuits due to its excellent orthogonality and robustness against diverse host backgrounds, including gram‐negative and positive bacteria and eukaryotes. To further leverage these advantages for multiplexed power supply, the transferability of orthogonal T7 RNAP variants, σ fragments, and other phage‐derived RNAPs like SP6 RNAP [12] should be evaluated. Integrating other components in the orthogonal central dogma [13], such as DNA polymerases, ribosomes, and tRNAs, into the power supply module will further isolate the heterologous genetic circuits from undesired interactions with host components (Figure 1B).

The signal processing capacity of the processor module will be unleashed by the interplay of universal parts operating at DNA, RNA, and protein levels, such as recombinases, transcription factors, riboswitches, riboregulators, ribozymes, proteases, and inteins. Recombinases excise or invert DNA sequences between their cognate recognition sites and have been harnessed to implement synthetic memory devices and complex logic circuits in diverse prokaryotes and eukaryotes, including plants [14]. Transcription factors such as CRISPR‐dCas systems and sigma factors have also shown cross‐species functionality in microbes like *Klebsiella oxytoca* [15] and *B. subtilis* [16], respectively. RNA‐based regulation relies on RNA interaction‐triggered conformation change to sequester or expose a regulatory element (usually ribosome binding sites or terminators), which is relatively conserved across the bacterial domain. The portability of RNA regulators has been demonstrated by theophylline‐responsive riboswitch, functional in over 12 bacterial species, including cyanobacteria [17, 18]. Recently, the broad host potential of riboregulators (small transcription activating RNA, STAR) [12] and ribozymes (split intron) [19] has also been reported. Inteins and proteases manipulate protein activities by associating or disassociating protein subunits, exhibiting functionality across bacteria, plant, and mammalian cells. Notably, an orthogonal library of split inteins has been established [20], and the split inteins have been coupled with other universal parts, namely sigma factors and TetR‐family repressors, for Boolean logic computation [20, 21]. Although only characterized in *E. coli*, the intein library and intein‐transcription factor hybrid circuits are theoretically universal and, thus, good candidates for transplantation to other hosts. Besides these genetic parts, their associated regulatory tools could also be valuable for tuning the universal circuits’ behavior. For instance, DNA sponges could be employed with transcription factors to reduce basal levels, mitigate toxicity, and generate a spectrum of the circuit’s response [22].

Next, standardized workflows need to be established to systematically evaluate the portability of genetic parts and compile them into databases. As discussed above, a set of genetic parts has been exhibited to be functional in multiple hosts. However, these demonstrations are qualitative, as the performances of these parts are reported using fluorescent readouts, and it can be tricky to define if a part functions in a host and how well its portability is. The host species, measurement units, and experimental procedures in different studies also vary, rendering the characterization data of these universal parts less comparable [3]. Quantitative parameterization will facilitate the capture of the cues about genetic parts’ universality by accessing the robustness of the critical parameters (e.g., Hill coefficients) against different host backgrounds. Establishing measurement standards and databases of universal parts with detailed descriptions of their functionality and transferability is also urgently needed to provide reusable and reliable resources for synthetic biologists to select parts for universal circuit design. Given that a curated part database, biopartsDB [3], has been available for *E. coli*, it could be upgraded by compiling the characterization data in other chassis, such as yeast, mammalian cells, and non‐model microbes.

Finally, enabling tools developed in model systems should be applied to hasten the DBTL cycles of genetic circuit design in non‐model systems. Tools for de novo design and sequence‐to‐function prediction of genetic parts will significantly expand the universal part toolbox with high‐performing, orthogonal, and non‐repetitive parts. For instance, NUPACK, a powerful platform to design RNA interactions, could be leveraged to devise orthogonal riboregulators and complex ribocomputing circuits [23] in non‐model microbes. Connecting universal parts into circuits could be aided by insulating parts from one another to avoid generating new parts during DNA assembly. Computational tools like R2oDNA Designer [24] could design biologically inactive DNA sequences as genetic spacers to prevent unwanted changes in gene expression due to genetic contexts. Integrating part design, selection, insulation, and concatenation will lead to design automation platforms transforming user‐defined specifications into DNAs encoding desired circuits. As transcriptional circuits designed by current algorithms like Cello [5] are over‐complicated for simple gates (e.g., AND gate), next‐generation platforms integrating multi‐level regulation to design genetically compact, less burdensome circuits should be developed. After successful deployment in novel host contexts, the universal circuits could be diagnosed by RNA sequencing [25] and optimized with active learning methods [26] and feedback optimizers [27].

Implementing universal genetic circuits in non‐model chassis requires the standardized characterization and quantitative parameterization of genetic part libraries in different host contexts. In this issue, Qin et al. [9] described such workflow to evaluate the universality of transcriptional modules extendable to other part categories and host organisms. This work represents a remarkable step toward the mission of predictively engineering universal circuits. In the future, expanding the regulatory toolbox, integrating computer‐aided design tools, and developing high‐throughput experimental pipelines will enable automatic design and effortless optimization of universal circuits to program non‐model organisms for delivering fieldable technologies and real‐world products.

## AUTHOR CONTRIBUTIONS

**Yuanli Gao**: Conceptualization; writing—original draft; writing—review and editing. **Baojun Wang**: Conceptualization; supervision; writing—review and editing.

## CONFLICT OF INTEREST STATEMENT

The authors Yuanli Gao and Baojun Wang declare no conflicts of interest.

## ETHICS STATEMENT

This manuscript does not involve a research protocol requiring approval by the relevant institutional review board or ethics committee.
